# Impact of interfacial coupling of oxygen octahedra on ferromagnetic order in La_0.7_Sr_0.3_MnO_3_/SrTiO_3_ heterostructures

**DOI:** 10.1038/srep40068

**Published:** 2017-01-11

**Authors:** Xiaoyan Li, Ionela Lindfors-Vrejoiu, Michael Ziese, Alexandre Gloter, Peter A. van Aken

**Affiliations:** 1 Stuttgart Center for Electron Microscopy, Max Planck Institute for Solid State Research, Heisenbergstr. 1, 70569 Stuttgart, Germany; 2 Laboratoire de Physique des Solides, CNRS UMR 8502, Université Paris Sud, 91405 Orsay, France; 3 Universität zu Köln, II. Physikalisches Institut, Zülpicher Str. 77, D-50937 Köln, Germany; 4 Universität Leipzig, Fakultät für Physik und Geowissenschaften, Abteilung Supraleitung und Magnetismus, Linnéstrasse 5, D-04103 Leipzig, Germany

## Abstract

La_0.7_Sr_0.3_MnO_3_, a half-metallic ferromagnet with full spin polarization, is generally used as a standard spin injector in heterostructures. However, the magnetism of La_0.7_Sr_0.3_MnO_3_ is strongly modified near interfaces, which was addressed as “dead-layer” phenomenon whose origin is still controversial. Here, both magnetic and structural properties of La_0.7_Sr_0.3_MnO_3_/SrTiO_3_ heterostructures were investigated, with emphasis on the quantitative analysis of oxygen octahedral rotation (OOR) across interfaces using annular-bright-field imaging. OOR was found to be significantly altered near interface for both La_0.7_Sr_0.3_MnO_3_ and SrTiO_3_, as linked to the magnetism deterioration. Especially in La_0.7_Sr_0.3_MnO_3_/SrTiO_3_ superlattices, the almost complete suppression of OOR in 4 unit-cell-thick La_0.7_Sr_0.3_MnO_3_ results in a canted ferromagnetism. Detailed comparisons between strain and OOR relaxation and especially the observation of an unexpected La_0.7_Sr_0.3_MnO_3_ lattice *c* expansion near interfaces, prove the relevance of OOR for the magnetic properties. These results indicate the capability of tuning the magnetism by engineering OOR at the atomic scale.

Manganites have been extensively studied for a quarter of a century as they display a strong coupling between spin, charge, lattice and orbital degrees of freedom, which not only leads to colossal magnetoresistance, but also to half-metallicity and new mechanisms of multiferroicity^1^,^2^,^3^. Beyond the interest in these bulk effects, interface engineering between manganites and other perovskites might specifically lead to new functionalities^4^. Since La_0.7_Sr_0.3_MnO_3_ (LSMO) is the ferromagnetic metal with highest Curie temperature (*T*_*C*_ ~ 370 K) among manganites and holds almost full spin polarization, it is generally used as a standard spin injector into heterostructures^5^,^6^. However, it has been realized early that the magnetic properties of LSMO and other manganites were strongly modified close to interfaces^7^. This was at first simply addressed as a dead layer^7^,^8^, but progressively more detailed models were developed. In our work, we focus on the La_0.7_Sr_0.3_MnO_3_/SrTiO_3_ (LSMO/STO) interface; in studies of LSMO thin films^9^,^10^,^11^ and LSMO/STO superlattices^12^,^13^,^14^,^15^,^16^,^17^ a recurring feature appears: below a certain critical thickness the LSMO layers become insulating and the ferromagnetic order is strongly reduced, but does not appear to vanish completely. The value of the critical thickness depends on extrinsic parameters such as strain^16^,^17^, oxygen content^17^,^18^, the presence of a capping layer^17^ and the laser fluence on the target^15^. The existence of a critical thickness, however, is not under debate; in a careful investigation of extrinsic parameters its minimum value has been put to a thickness of 3 unit cells (u.c.)^17^.

On the other hand, the origin of this dead layer is still under debate: it might be due to structural imperfections such as intermixing at interfaces and variations in the A-site cation ratio^15^ as well as oxygen vacancies^18^; indeed, it is difficult to maintain the La/Sr ratio in a 2–3 u.c. thick film adjacent to STO^19^; as an alternative, the breakdown of ferromagnetism and metallic conductivity was attributed to the effect of strain and tetragonal distortions leading to spin canting and ultimately antiferromagnetic order^13^,^20^,^21^; as a further alternative, phase separation into coexisting antiferromagnetic insulating and ferromagnetic metallic mesoscopic regions was proposed^22^,^23^; moreover, interfacial orbital reconstruction might occur destroying the double-exchange interaction close to the interface^24^,^25^,^26^; additionally, there might be interfacial charge transfer^18^,^27^,^28^,^29^. The intimate coupling between metallic conductivity and ferromagnetism that is implied by the double exchange mechanism^30^ could even be absent in very thin layers, since intrinsic defects such as cation disorder lead to the localization of charge carriers^1^,^17^. In this work we want to highlight oxygen octahedral rotations as another mechanism that might be essential in understanding the physics of manganite interfaces.

In LSMO/STO based heterostructures, both LSMO and STO have ABO_3_ type perovskite structure, but due to different tolerance factors, STO is cubic at room temperature with a transition to a tetragonal phase below 105 K, whereas LSMO is rhombohedral in bulk^31^, but orthorhombic/tetragonal in thin films^32^,^33^. This demonstrates that the perovskite structure is versatile in accommodating chemical strain, as well as external structural strain. Another important parameter for structural accommodation is the rotation of the BO_6_ oxygen octahedra, which might especially occur at chemically sharp and coherent interfaces between two perovskites with differences in lattice constant and tolerance factor. Recent studies report that changes of BO_6_ oxygen octahedra rotation play a decisive role in determining the magnetocrystalline anisotropy^21^,^34^,^35^,^36^.

In this letter, we characterize the structural reconstruction at LSMO/STO interfaces by mapping the BO_6_ oxygen octahedral rotation and lattice parameters quantitatively. To this end we use state-of-the-art annular bright field (ABF) imaging in a Cs-corrected scanning transmission electron microscope (STEM), in the [1–10] zone axis orientation. We found an unexpected out-of-plane lattice evolution near the interfaces which evidenced that the OOR mismatch, instead of strain, primarily governs the interface structure. An OOR mismatch of ~10° across the LSMO/STO hetero-interface leads to a significant alteration of the rotation angles in both STO and LSMO layers, such that the suppression of OOR in LSMO can be intuitively linked to the deterioration of magnetism. The almost complete suppression of OOR in 4 u.c. thick LSMO within a LSMO/STO superlattice results in a strongly canted magnetism, which discloses the previously reported magnetic-dead-layer phenomenon. These findings suggest an efficient interfacial engineering mechanism of magnetic and electronic properties through OOR coupling at the atomic scale.

## Results

### Magnetic properties and thin film morphologies

Epitaxial thin films were fabricated by pulsed laser deposition from La_0.7_Sr_0.3_MnO_3_ stoichiometric polycrystalline targets onto SrTiO_3_ (100) substrates. Two representative samples were selected: (1) a triple layer sandwich-like sample (denoted as T2 in the following) consisted of two LSMO layers with thicknesses of 10 and 20 u.c. spaced by a 3 u.c. thick STO interlayer; (2) a LSMO/STO superlattice (denoted by SL15 in the following) consisting of 15 bilayers of 4 u.c. thick LSMO and 6 u.c. thick STO. Summary and comparison of the Curie temperatures for LSMO/STO heterostructures with different LSMO thickness can be found in supporting information Figure S1.

**Figure 1:**
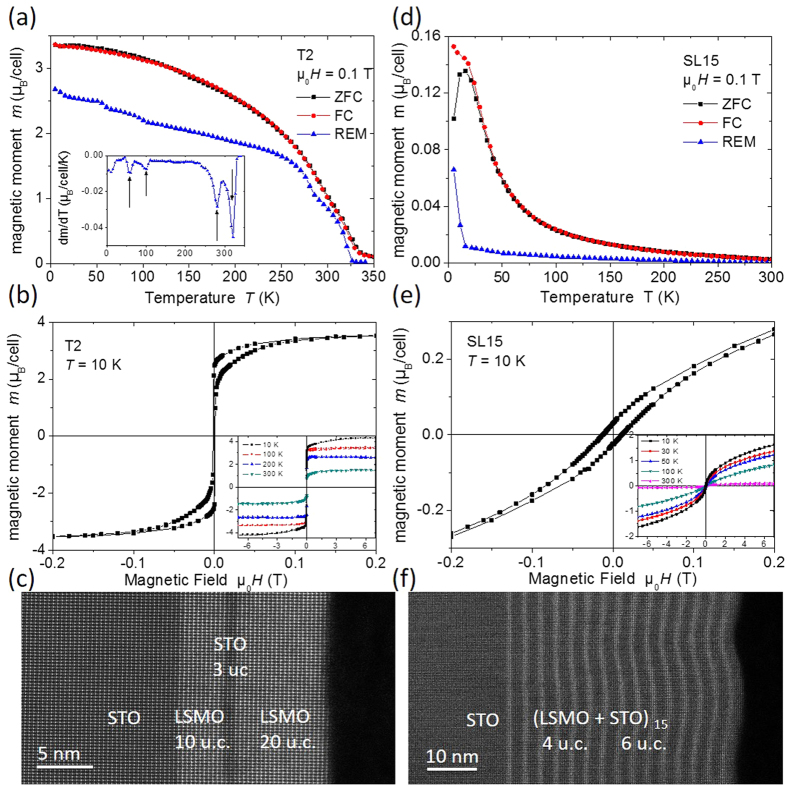
Left: trilayer sample T2, right: superlattice SL15. (**a**) and (**d**) Magnetic moments as a function of temperature during zero-field cooling (ZFC), field cooling (FC) and the remanent magnetization (REM). The inset in (**a**) shows the derivative of the remanent magnetization to highlight the magnetic transitions. (**b**) and (**e**) Magnetic moments as a function of applied field. The insets show the magnetic hysteresis loops up to 7 T at various temperatures. (**c**) and (**f**) Low magnification STEM-HAADF images.

Figure 1 shows the magnetic response of the two samples as well as their low resolution STEM-HAADF images. In Fig. 1(a) and (d) the magnetic moment was measured in an in-plane applied field of 0.1 T after zero-field cooling (ZFC), during field cooling (FC) and after setting the field to zero at 5 K, i.e. in the remanent (REM) state. Trilayer sample T2 showed a sizable spontaneous magnetic moment; four transitions were observed as a function of temperature as highlighted by the derivative in the inset of Fig. 1(a). The two high temperature transitions at 280 K and 320 K are the Curie temperatures of the 10 and 20 u.c. thick LSMO layers, respectively. A third transition at 100 K is attributed to the cubic-to-tetragonal transition of the STO substrate that distorts the layers and via the magnetoelastic coupling leads to a modification of the magnetic anisotropy^37^. The origin of the fourth transition at 60 K is less clear; it is reminiscent of a surface spin-glass transition reported for LSMO nanoparticles^38^; we will comment on this feature further in the conclusions. Figure 1(b) shows a ferromagnetic hysteresis loop at 10 K, with hysteresis loops up to 7 T at various temperatures in the inset. The sample is a soft ferromagnet with a coercive field of 1.3 mT at 10 K; the magnetic moment per Mn is (3.6 ± 0.2) μ_B_/unit cell, in good agreement with the spin-only moment of 3.7 μ_B_/unit cell. In contrast, superlattice sample SL15 does not show a sharp ferromagnetic transition, but a gradual onset of magnetic order approximately around 55 K, see Fig. 1(d); this is reminiscent of a disordered ferromagnet or a spin glass. The magnetic moment is 1.5 μ_B_/unit cell at 10 K and 7 T, see inset to Fig. 1(e), but the magnetization was not saturated in that field, which is characteristic of a canted ferromagnetic state^13^. The coercive field was enhanced to 12 mT at 10 K. Overall the magnetic properties of the present samples are very similar to corresponding data reported in the literature^10^,^12^,^13^,^14^,^15^,^16^,^17^.

The STEM-HAADF images in Fig. 1(c) and (f) show that the LSMO/STO heterostructures have been found coherent and defect free, with no apparent formation of misfit dislocations at the interfaces. In these Z-contrast HAADF images, the brighter regions corresponded to the heavier LSMO layers and the darker regions to STO layers. The well-defined continuous sub-layers and homogenous image contrast indicate the absence of phase separation or strain segregation along the in-plane direction. In the trilayer sample T2, the interfaces were found to be straight with an irregularity of only 1 u.c. along a distance of a few hundred nm. In the LSMO/STO superlattice SL15, as shown in Fig. 1(f), there is considerable curvature of the interfaces with a period of about 20 nm due to the elastic strain relaxation^39^.

### Quantitative OOR and strain characterization

The oxygen octahedra network was investigated with an atomically resolved ABF imaging technique which is capable to image both heavy cations and oxygen ions simultaneously^40^. Most of the previous ABF observations of oxygen octahedra in perovskite manganite layers were made in the <100>_pc_ zone axis orientation, where the linear density of the oxygen columns is given by 1/*a*, the same as the neighboring A/B cation column; *a* denotes the lattice parameter of the pseudo-cubic perovskite structure, further indicated by the subscript *pc*. Therefore, the signal from the oxygen columns is weak and masked by the strong contrast from the heavier A/B columns such that it is not easily visible. However, along the <110>_pc_ zone axis the linear density of the oxygen columns increases up to ~1.4/a, whereas the density of the A/B cation columns decreases to ~0.7/a. These atomic density changes boost the enhancement of the relative oxygen/cation contrast; therefore, under the premise of high spatial resolution in a Cs-corrected STEM, the <110>_pc_ zone axis provides a better contrast of the oxygen columns than the <100>_pc_ zone axis. Moreover, considering the characteristics of in-plane oxygen octahedral rotation, such as in the case of *a*^−^*a*^−^*c*^+^ (following Glazer’s notation^41^) in orthorhombic LSMO (space group *Pnma*)^42^, the anti-phase rotation in the <100>_pc_ zone axis introduces a split of the oxygen columns, thus blurring and eclipsing the oxygen atomic propagation, while the in-phase rotation in the [1–10]_pc_ zone axis gives a sharp propagation. Accordingly, the [1–10]_pc_ zone axis was selected in our work for an enhanced contrast of the oxygen and in-phase octahedral rotation in each LSMO monolayer of the heterostructures (a detailed quantitative ABF intensity analysis can be found in the supporting information Figure S2).

**Figure 2:**
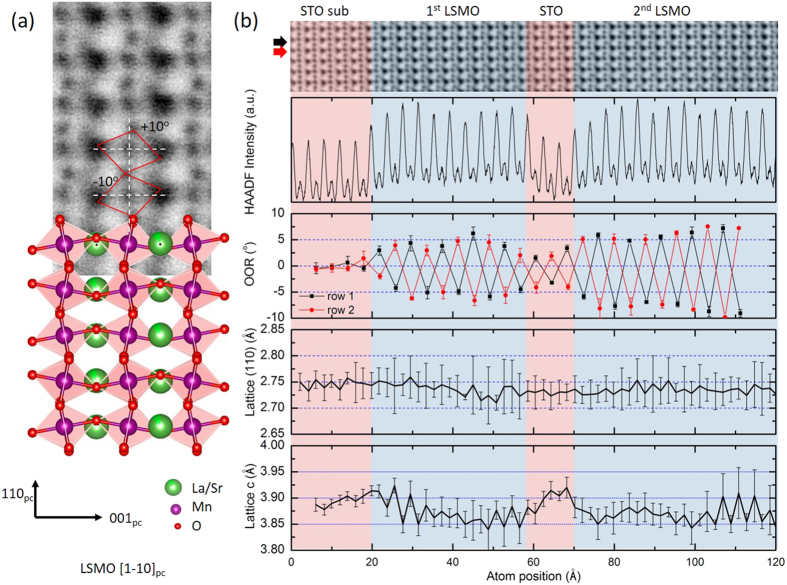
(**a**) STEM-ABF image of LSMO taken at the 13–14th monolayer of the top LSMO layer of the trilayer T2 sample. All atoms are imaged as dark spots and could be fitted with a bulk-like LSMO structure model shown below. (**b**) From top to bottom: ABF-STEM image of the sample T2 showing both the STO substrate and the LSMO-STO-LSMO heterostructure, the intensity profile of the corresponding HAADF image, the oxygen octahedral rotation angle, and the lattice spacing along the [110] _pc_ axis and the out-of-plane *c* axis. The STO substrate and the STO center layer is shadowed in red and the LSMO layers are shadowed in blue. The line profiles are the average values of all atomic rows presented in the ABF image and the error bars are the standard deviation using the formulation 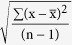.

A typical STEM-ABF image of LSMO in the [1–10]_pc_ zone axis orientation obtained from the 13^th^–14^th^ monolayer of the 20 u.c. thick top LSMO layer of sample T2 is shown in Fig. 2(a). The imaged area is located relatively far from the highly strained hetero-interface, which can be considered as strain-free and to describe a structure close to bulk LSMO. The oxygen atom columns are clearly visualized in the images with a contrast as high as ~60% of the Mn columns at a spatial resolution of 70 pm. The atomic model for the orthorhombic LSMO structure (*Pnma*) with MnO_6_ tilt of *a*^−^*a*^−^*c*^+^ is fitted into the ABF image. In this zone axis, the in-phase rotation of the oxygen octahedra along the observation direction yielded a sharp oxygen atomic column in the image. A clear zig-zag pattern of the oxygen atoms can be seen along the [110]_pc_ direction which can be used to determine precisely the oxygen octahedral rotation, which is ±10°, close to the value for the bulk structure. The definition of the oxygen octahedral rotation (OOR) angle is sketched in the ABF image with a clockwise-rotation defined as positive.

A representative STEM-ABF image taken across the substrate and the three sublayers of the trilayer T2 is shown at the top of Fig. 2(b), following the intensity profile of its corresponding HAADF image which is roughly proportional to Z^1.7–2^. Therefore, the pronounced peaks in the HAADF intensity image mark the position of the heavy ions, whereas the oxygen atomic columns are not visible in the HAADF image. According to the HAADF intensity profile, two different components, STO (substrate and middle layer) and LSMO, are clearly distinguished and marked with red and blue shades, respectively. Atomically sharp interfaces were observed for the thin films terminated with the La/SrO plane and an intermixing of Ti and Mn limited to 1 u.c. Average values, over all the atomic rows along the in-plane direction, of the OOR angles and the lattice parameters are shown in the last three panels of Fig. 2(b). As expected from the structural characteristics, approximately equal octahedral rotations, but with opposite signs, were found in the OOR profiles of two neighboring atomic rows inside the LSMO layers; these angle values oscillate along the growth direction, whereas the rotation angles were close to zero within the cubic STO substrate. The accommodation of the oxygen octahedral mismatch of 10° between LSMO and STO results in a significant modification of the octahedral rotation of both LSMO and STO near the interfaces. An obvious reduction of the OOR within the first few unit cells of LSMO adjacent to the strained interfaces was found; this gradually relaxed towards the cores of the LSMO layers. By comparing the two LSMO layers, the interfacial oxygen octahedral coupling behavior could be revealed. The 10 u.c. thick LSMO layer is constrained on both sides by STO and only reached a maximum OOR angle of 5° in the middle, forming a plateau 6 u.c. thick. The top 20 u.c. thick LSMO layer is constrained only by the STO interlayer that in turn is also greatly constrained and distorted, thus the OOR instantly reaches a strained plateau of around 6° and remains there for a transition thickness of 5 u.c. before increasing within the next 2 unit cells to the bulk-like value of 9°.

Whereas the OOR is suppressed on the LSMO side of the interface, it was found to be induced on the STO side, where it follows the oxygen octahedra rotation pattern of LSMO, giving rise to a clearly visible in-plane tilt of 2° within the first 2 u.c. of the substrate and of 3° across the STO interlayer. This induced rotation decays within 4 u.c. in the substrate, indicating a shorter impact length of the interfacial coupling in STO as compared to LSMO. Consequently, near LSMO/STO interfaces the OOR is modified to accommodate the oxygen octahedral rotation mismatch with material dependent propagation lengths of 4 u.c. in STO and 7 u.c. in LSMO.

The corresponding lattice parameters along the in-plane [110]_pc_ direction, i.e. parallel to the LSMO/STO interface, and along the [001]_pc_ growth direction, i.e. perpendicular to the LSMO/STO interface, are shown in the last two panels of Fig. 2(b); the lattice parameter of the STO substrate is used for calibration and is set to 0.3905 nm. The coherent epitaxial growth of LSMO on STO is expected to result in a tensile in-plane strain of 0.7% in LSMO layer. However, the in-plane and out-of-plane lattice constants of the film show different strain relaxation behavior. Within the first 3 unit cells, the in-plane lattice parameter of the 10 u.c. thick LSMO layer was highly strained and identical to the substrate lattice parameter before relaxing to the LSMO bulk value at a distance of 5 u.c.; it then stayed constant through the remaining part of the thin film. As compared to the OOR evolution, the in-plane lattice had a similar highly-strained region next to the interface, but a shorter relaxation length without metastable plateau behavior. Notably, the STO interlayer was highly strained by the two neighboring LSMO layers, and adapted the LSMO in-plane lattice constant. On the other hand, the out-of-plane lattice exhibited a higher degree of freedom than the in-plane lattice, showing a relaxation length of only 3 u.c. for LSMO and 1 u.c. for STO; such a relaxation length is further shortened in the 20 u.c. thick LSMO layer to only 1 u.c., probably because the strain by the interlayer is less than by the substrate. Interestingly, a small lattice expansion along the *c* axis of about 0.6% was found at the STO (substrate)-LSMO interface.

According to our observations, the mechanisms for accommodating lattice mismatch and oxygen octahedra rotation mismatch, although being linked to each other, are different. The magnetic properties are certainly related to both the strain and octahedral rotation state. The magnetic data show a clear difference in the Curie temperatures of 280 K and 320 K of the 10 and 20 u.c. thick LSMO layers with identical transition widths. Since the strain states of the two LSMO layers were mainly identical, we argue that the Curie temperature difference is due to the OOR differences with a metastable plateau state in the 10 u.c. and a fully relaxed bulk-like state in the 20 u.c. layer. In view of the suppressed magnetization in the superlattice, the 60 K transition observed in the trilayer might be related to the interfacial LSMO regions with strongly decreased OOR.

**Figure 3:**
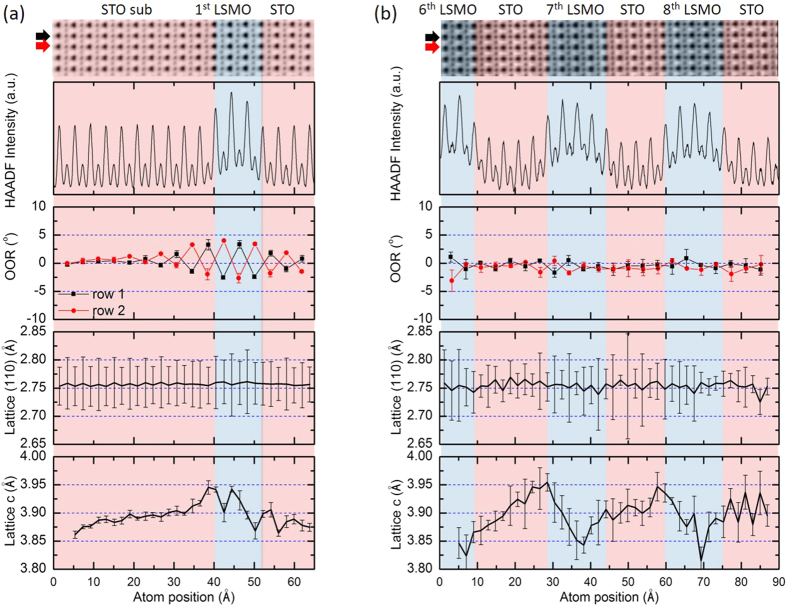
From top to bottom. STEM-ABF images, the intensity profile of the corresponding HAADF image, the oxygen octahedral rotation angle, the lattice spacing along the [110]_pc_ and c axis, for (**a**) the first layer of LSMO and (**b**) LSMO layers from the middle of superlattice SL15. The STO substrate (STO sub) and STO layers are shadowed in red and the LSMO layers are shadowed in blue. The line profiles are the average values of all atomic rows presented in the ABF image and the error bars are the standard deviation using the formulation 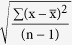.

We performed the same quantitative atomic analysis for the LSMO-STO superlattice sample SL15. In Fig. 3, STEM-ABF images obtained from two representative regions of SL15 are shown: the left panel is focused on the interfaces between substrate, first LSMO layer and first STO layer, the right panel is focused on the central part of the superlattice. The STO and LSMO layers were distinguished by the corresponding HAADF intensity profiles and marked by red and blue shades. The graphs in the third panel of Fig. 3 show the oxygen octahedral rotation profiles: in the first LSMO layer octahedra rotations were still observed, but - compared to the bulk or thick film - with a reduced rotation angle of 4°; this is similar to the first two unit cells of the first LSMO layer in the trilayer sample, see Fig. 2(b). The induced octahedra rotations in the STO substrates behave the same as in the trilayer sample with the first 2 u.c. strongly affected by the neighboring LSMO oxygen octahedra rotation. Contrary to these findings, in the other LSMO layers that are sandwiched between the thin STO layers, the oxygen octahedral rotation is almost completely suppressed. This suppression of the OOR in LSMO might be concomitant to the generally found canted magnetism in such kind of superlattice.

The lattice relaxation of SL15 is shown in the last two panels in Fig. 3. The in-plane lattice constant along [110]_pc_ was clamped to the STO substrate lattice constant which is commonly found in superlattice structures. However, the lattice parameter *c* along the growth direction showed a clear oscillation with the bilayer period. A lattice expansion of around 1.3% was found at all interfaces which is approximately double of that observed in the trilayer sample, indicating a stronger interfacial constraint. In particular, this is clearly visible at the first LSMO layer next to the substrate. The *c* expansion starts at the last STO unit cell, but also propagates into the first two unit cells of the LSMO layer. Such an expansion of the LMSO *c* lattice constant is an unusual behavior, since an out-of-plane compression of LSMO is expected for the tensile strain at the STO/LSMO interface. Previous coherent Bragg rod analysis and synchrotron X-ray diffraction have also indirectly reported the occurrence of a *c* expansion for the first 2–3 unit cells of LSMO thin film growth on STO^21^,^43^. This unexpected expansion indicates that strain is not primarily governing the LSMO structural properties at the interface, but that OOR mismatch is the significant factor. It is noteworthy that this lattice *c* oscillation also results in an inhomogeneous local tetragonality within the LSMO layers. Moreover, the chemical analysis across the interfaces were also shown in the supporting information Figure S3. A small reduction of Mn valence can be found near the interfaces within 1 u.c., indicating a very weak and located charge transfer effect which might only play a limited role in the interfacial reconstruction.

## Discussion

The exact origin of the canted magnetism in LSMO/STO superlattices is difficult to assess. In the manganites, the *T*_*c*_ is proportional to the electronic bandwidth *W*^1^,^44^; the latter depends on the Mn-O bond distance *d* and the Mn-O-Mn bond angle *ϕ: W* = cos[* (π-ϕ)/2]/d*^*3.5* ^45,46. In case of the superlattice the bond angle *ϕ* decreases with the suppression of the octahedral rotations yielding the paradoxical result that the ferromagnetism should actually be enhanced. A change of the *e*_*g*_ population due to biaxial strain and concomitant tetragonality was also reported to reduce *T*_*c*_, with a typical rate of 10% for a tensile strain of 1%^47^. However, such a strain induced *e*_*g*_ population change cannot explain the magnetic properties of the superlattice, especially because the STEM analysis showed inhomogeneous tetragonality and a resulting average *c/a* ratio close to 1.

On the other hand, the magnetic canting seems to be primarily related to the suppression of the OOR that occurs partially nearby the interface of sample T2 or completely in sample SL15. Previous work has reported the occurrence of a spin glass constrained at the interface between LSMO and STO^48^,^49^. Monte-Carlo simulations based on the double exchange model gave also evidence that the OOR plays a more important role than the dimensionality effect for the superlattice and might result in canted magnetism^50^. These findings stress the role of the OOR at the interface for the physical properties.

Recent work has shown that for LSMO layers, the magnetic easy axis might be strongly altered by the local OOR^35^. In the case of the superlattice, the OOR suppression might result in a frustrated Mn spin configuration close to the interface. The occurrence of a strong population change at the interface was also reported by XLD measurements^51^,^52^,^53^. While thick LSMO grown on STO substrate has negative XLD signal indicating a preferential *x*^*2*^*-y*^*2*^ occupation as expected for tensile strain, very thin film (4 u.c.) has a very small preferential *3z*^2^*-r*^*2*^ occupation^51^. It was concluded that close to surface and interface, the Mn *3d e*_*g*_ *3z*^*2*^*-r*^*2*^ orbital is preferentially occupied and the role of local strain (that favors respectively *3z*^*2*^*-r*^*2*^ or *x*^*2*^*-y*^*2*^ for compressible or tensile strain) is a secondary effect^51^,^52^,^53^. The strong suppression of the in-plane OOR, irrespective of the local *c/a* ratio, can be the origin of a favored *e*_*g*_ *3z*^*2*^*-r*^*2*^ population. The microscopic origin of such population change is difficult to elucidate. The OOR suppression can play a role e.g. by altering the electron-phonon coupling leading to Jahn-Teller effect. It is also possible that the heterogeneous c/a might actually average to a slightly compressive situation that favor a slightly *e*_*g*_ *3z*^*2*^*-r*^*2*^ population. This will be in agreement with the very weakly positive XLD signal observed for thin LSMO on STO^51^. In any case, such population change favors the formation of C-type AF ordering at the interface^50^ and might also participate in the magnetism canting for the SL15 sample.

Thus, the evolution of the OOR in LSMO is expected to be the primary mechanism for the thickness-driven magnetism deterioration. The oxygen octahedral mismatch shows impact on both sides of the interface, depending on the elastic properties of the oxygen network; its relaxation length can differ greatly for different materials. In case of the LSMO/STO thin film, relaxation lengths of 4 u.c. for STO and 7 u.c. for LSMO were found. The general dead-layer behavior in LSMO/STO superlattices for LSMO layer thicknesses below 3 u.c., can then be predominantly attributed to a large deviation of the OOR from the bulk value. Therefore, to achieve a high TMR ratio in a LSMO-STO heterostructure, a critical thickness of LSMO of at least 7 u.c. (3 u.c. buffer to each interface) is required for a moderate ferromagnetism, and a critical thickness of STO of at least 5 u.c. (2 u.c. buffer to each interface) for a good insulating barrier layer. These values are in good agreement with previous reports^17^,^54^.

In summary, the quantitative profiles of the lattice parameters and OOR crossing the coherent interfaces of LSMO/STO heterostructures with relatively thick (larger than 10 u.c.) and ultrathin LSMO layers (4 u.c.) were determined based on STEM-ABF imaging with sub-Å precision. A significant variation of the OOR was found near the interface to accommodate the rotation angle mismatch of 10° between LSMO and STO, which propagates over the LSMO and STO structure for 7 u.c. and 4 u.c., respectively. The suppression of the OOR observed in LSMO was argued to be related to the dramatic thickness-dependent deterioration of the magnetic properties. Especially, in the LSMO/STO superlattice, the almost complete suppression of octahedral rotation in 4 u.c. thick LSMO leads to a strongly canted magnetism, which is in good agreement with other reports and explains the “dead-layer” phenomenon. An unexpected out-of-plane lattice expansion within the LSMO layers near the interfaces further stresses the primary role of OOR mismatch on the interface structural reconstruction. These results indicate the capability of tuning the magnetism by engineering the oxygen octahedral rotation on the atomic-scale with different film thicknesses and adjacent oxygen networks. The strong coupling between the oxygen octahedral rotation and functionalities sheds light on both fundamental research and practical spintronic device design.

## Methods

### Thin film deposition

Epitaxial thin films, trilayers and superlattices were fabricated by pulsed laser deposition (KrF laser, 248 nm) from La_0.7_Sr_0.3_MnO_3_ stoichiometric polycrystalline targets onto SrTiO_3_ (100) substrates. The STO substrates were etched in buffered HF and annealed at 1000 °C for 2 hours in air, in order to obtain substrates with atomically flat terraces and TiO_2_-termination. Substrate temperature and oxygen partial pressure during film growth were 700 °C and 0.14 mbar O_2_, respectively. The growth of thin film was *in-situ* monitored by a reflective high energy electron diffraction (RHEED) system, thus giving precise control on the thickness down to unit cell scale.

### Magnetization measurement

Magnetization measurements were done with a Quantum Design SQUID magnetometer under varied temperature and magnetic field. The measurements were performed with the following cycle which is typical for the study of spin glasses or high temperature superconductors:

1. ZFC: demagnetizing the sample at 300 K, cooling in nominal zero field down to 5 K, stabilizing the temperature at 5 K, setting the magnetic field to the required value in no-overshoot mode and measuring during warmup.
2. FC: directly after the ZFC run, measuring during cooldown in the same field as in the ZFC measurements.
3. REM: After stabilizing a temperature of 5 K at the end of the FC run, the magnetic field was reduced to zero in no-overshoot mode and the remanent magnetic moment measured during warmup.

### Transmission electron microscope characterization

Cross-section specimens for electron microscopy study were prepared along the [1–10]_pc_ zone axis of the STO substrate by standard tripod polishing, followed by Ar-ion milling under liquid nitrogen cooling (PIPS II, Gatan, USA). The microstructure was investigated in a JEOL ARM 200CF scanning transmission electron microscope (STEM) with a probe Cs corrector, utilizing atomically resolved high angle annular dark field imaging (HAADF, collection angles 115–276 mrad), and annular bright field imaging (ABF, collection angles 11–23 mrad) which enables to directly image oxygen atoms in real space and to map a projection of the oxygen octahedral network. These imaging experiments were performed at an acceleration voltage of 200 kV, with a semi-convergence angle of 21 mrad, showing a typical spatial resolution of 70 pm. In order to diminish the sample drift effect, before starting the image acquisition the electron beam was blocked for at least 5 minutes. The typical scanning step for atom resolution images was 0.06 Å/pixel with a dwell time of 16 μs/pixel, and the scanning direction is perpendicular to the interfaces. The corresponding interfacial chemical distribution was studied by electron energy loss spectroscopy (EELS).

## Additional Information

**How to cite this article:** Li, X. *et al*. Impact of interfacial coupling of oxygen octahedra on ferromagnetic order in La_0.7_Sr_0.3_MnO_3_/SrTiO_3_ heterostructures. *Sci. Rep.* **7**, 40068; doi: 10.1038/srep40068 (2017).

**Publisher's note:** Springer Nature remains neutral with regard to jurisdictional claims in published maps and institutional affiliations.

## Supplementary Material

Supporting Information
