# Supplementary material for: Impact of interfacial coupling of oxygen octahedra on ferromagnetic order in La0.7Sr0.3MnO3/SrTiO3 heterostructures
Source: Sci Rep. 2017 Jan 11;7:40068. doi: 10.1038/srep40068 (PMC5225431; doi:10.1038/srep40068)
Supplement: Supporting Information [file srep40068-s1.pdf]

Supporting Information

# Impact of interfacial coupling of oxygen octahedra on ferromagnetic order in $La_{0.7}Sr_{0.3}MnO_3/SrTiO_3$ heterostructures

*Xiaoyan Li,[1,4,*] Ionela Lindfors-Vrejoiu,[2] Michael Ziese[3], Alexandre Gloter[4] and Peter A. van Aken[1]*

[1] Stuttgart Center for Electron Microscopy, Max Planck Institute for Solid State Research, Heisenbergstr. 1, 70569 Stuttgart, Germany

[2] Universität zu Köln, II. Physikalisches Institut, Zülpicher Str. 77, D-50937 Köln, Germany

[3] Universität Leipzig, Fakultät für Physik und Geowissenschaften, Abteilung Supraleitung und Magnetismus, Linnéstrasse 5, D-04103 Leipzig, Germany

[4] Laboratoire de Physique des Solides, CNRS UMR 8502, Université Paris Sud, 91405 Orsay, France

1. Comparison of Curie temperature for different LSMO/STO heterostructures

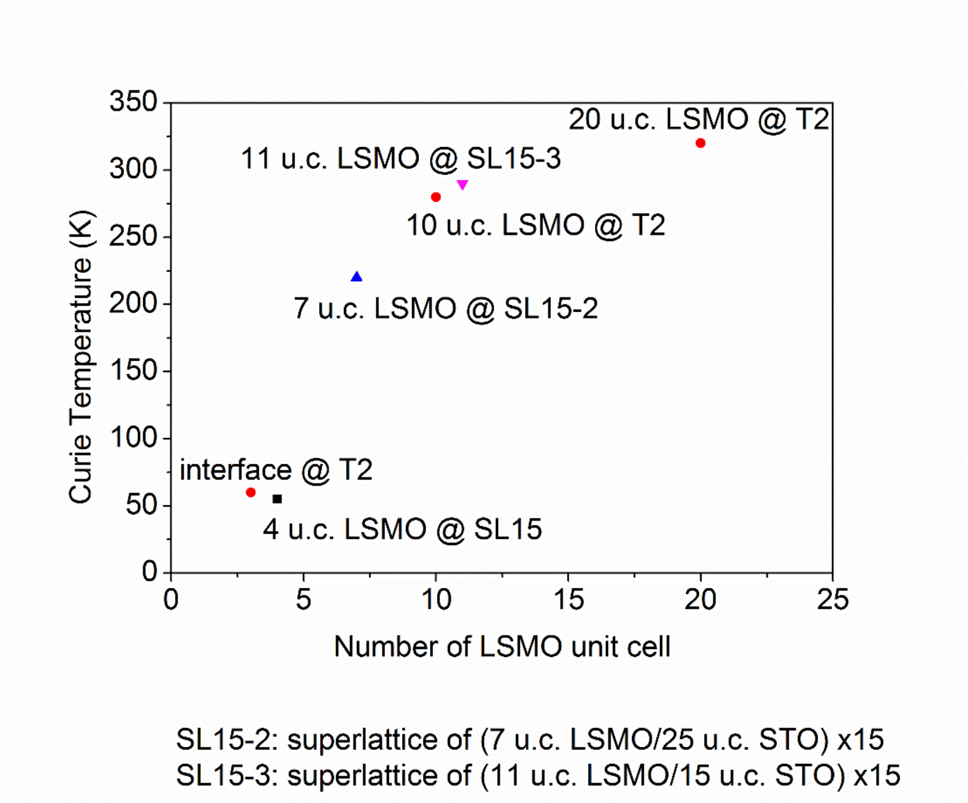


**Figure S1** Curie Temperatures of LSMO/STO heterostructures as a function of the thickness of LSMO layer: including two representative samples discussed in the main text: trilayer sample T2 and superlattice sample SL15, and two other superlattice samples SL15-2 and SL15-3 with 7 u.c. and 11 u.c. LSMO layer respectively.

Figure S1 shows the curie temperature of LSMO/STO heterostructures as a function of the thickness of LSMO layers, ranging from 20 u.c. to 4 u.c.. The curie temperature decreases as the thickness of LSMO layer is reduced, and finally display a dead-layer behavior at a thickness of 4 u.c., which is in good agreement with previous reports.

2. Quantitative STEM image calculations:

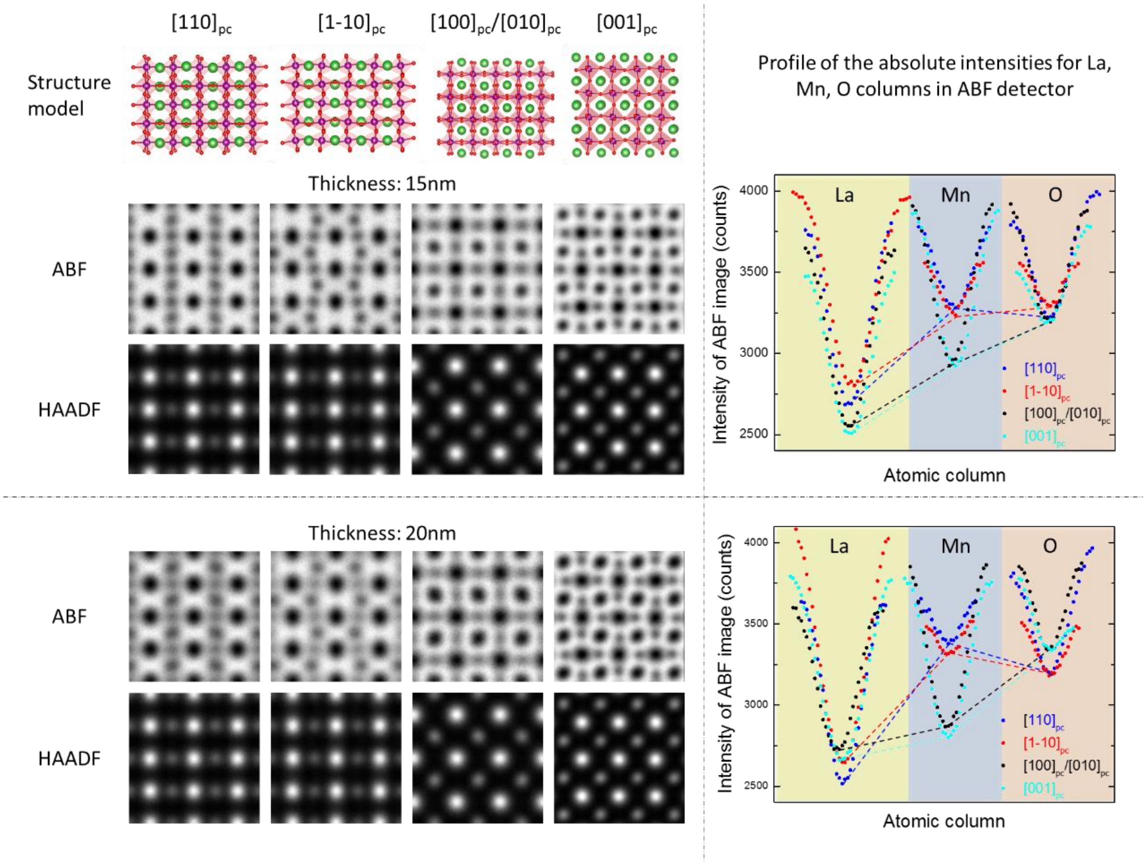


**Figure S2** Quantitative STEM-ABF and HAADF image calculations using QSTEM[1] of orthorhombic LSMO in <110>$_{pc}$ and <100>$_{pc}$ zone axis orientation, at the typical thickness of 15 nm and 20 nm, respectively. The subscript *pc* stands for pseudo-cubic setting.

In order to choose the best crystal orientation for the oxygen octahedral observation in orthorhombic LSMO structure of $a^-a^-c^+$ rotation characteristic (*Pmna*, space group number 62, $a_{pc}$ = 3.875 Å), the STEM images of different zone axis are calculated quantitatively using the QSTEM framework[1] with the parameters obtained from a typical experimental setup for the JEOL-ARM200CF with a probe-corrector: high voltage 200 kV, convergence angle 20 mrad, residual Cs parameter of 0.005 mm, Scherzer focus of -4.3nm, probe size 0.8 Å, source brightness $5*10^8$ A/cm$^2$sr, HAADF collection angles between 120-200 mrad, ABF collection

angles between 11-22 mrad, and run for 10 TDS, image dwell time 15μs/pixel with Poisson noise added.

All the low-index zone axis of the cross-sectional TEM sample for the heterointerface observation are considered. There are two inequivalent $<110>_{pc}$ directions and two equivalent $<100>_{pc}$ directions. Their corresponding structure models are presented in Fig. S1 top row as references. Notably, $[001]_{pc}$ is the growth direction of the film, which cannot be used for the interface investigation, however, as a typical orientation with an in-phase rotation pattern, it is also presented here for comparison. The ABF images at two typical TEM sample thicknesses of 15 nm and 20 nm are calculated and listed in Fig. S1 left plane under their structure models, as well as their corresponding HAADF images as references. Generally, ABF images at the thickness of 15 nm give better contrast between oxygen and heavy cation columns with sharper atom projection. Therefore, a thinner sample is always recommended for a better resolved ABF imaging. As compared with the experimental results in the main text for the [1-10] zone axis, the thickness of our sample can be estimated to be around 15 nm. The best observation zone axis is firstly considered regarding the different oxygen octahedra rotation characteristic at different orientations. In LSMO, due to the anti-phase oxygen octahedral rotation along the $[110]_{pc}$ and $[100]_{pc}/[010]_{pc}$ axis, the oxygen atoms are not aligned but split into two columns. Since the projected separation of the oxygen atoms is so small (~0.6Å) that it cannot yet be resolved even with a state-of-art STEM, and appears as an elliptical shape which is not conducive for a precious determination of oxygen atom position. However, along the $[1\text{-}10]_{pc}$ zone axis, the oxygen atoms are aligned under the in-phase octahedral rotation which gives a sharp circular oxygen atomic propagation and a high accuracy for determining the oxygen position and the $BO_6$ rotation angle. Thus in our case, the $[1\text{-}10]_{pc}$ zone axis is chosen for the oxygen observation.

It is noteworthy that, during the experiments, we found it always easier to visualize the oxygen atoms at the $<110>_{pc}$ zone axis than $<100>_{pc}$. In order to understand this phenomenon which would be practically useful for ABF studies, a quantitative comparison of the ABF imaging intensity profile for La, Mn, O atomic columns are also shown in Fig. 1S right plane for each thickness. Considering the perovskite atomic structure, the density of oxygen column is 1/a at the $<100>_{pc}$ axis (a is the lattice parameter of the pseudo-cubic structure), the same as the A/B site columns. However, at the <110>pc zone axis, the oxygen density rises up to ~1.4/a while the density of A/B cation column decreases to ~0.7/a, indicating a better oxygen contrast. Unlike the Z-contrast HAADF imaging, the contrast formation in ABF imaging is a complex combination of phase-contrast and mass-thickness contrast, thus the propagation intensity of the atom columns is only partially related to the atom density of column. In our particular case, from the quantitative profile of each element columns, one can extract that the heavy La (Z=57) column doesn’t change much with the change of thickness or orientation. Meanwhile, the intensity of Mn columns change apparently with orientation, exhibiting a smaller intensity (less dark) in $<110>_{pc}$ zone axis orientation. The oxygen columns don’t differ much between the two orientations at the thickness of 15 nm, however, the difference does show up at a thickness of 20 nm with an enhanced intensity for the $<110>_{pc}$ zone axis. Therefore, the oxygen contrast as referred to its neighboring Mn columns is greatly improved in $<110>_{pc}$ zone axis orientation and further strengthens with the increase of thickness, which is probably due to channeling effects along and between Mn and O columns. Thus, as a general rule, $<110>_{pc}$ is a better axis for oxygen observation.

3. EELS analysis of the LSMO-STO superlattice

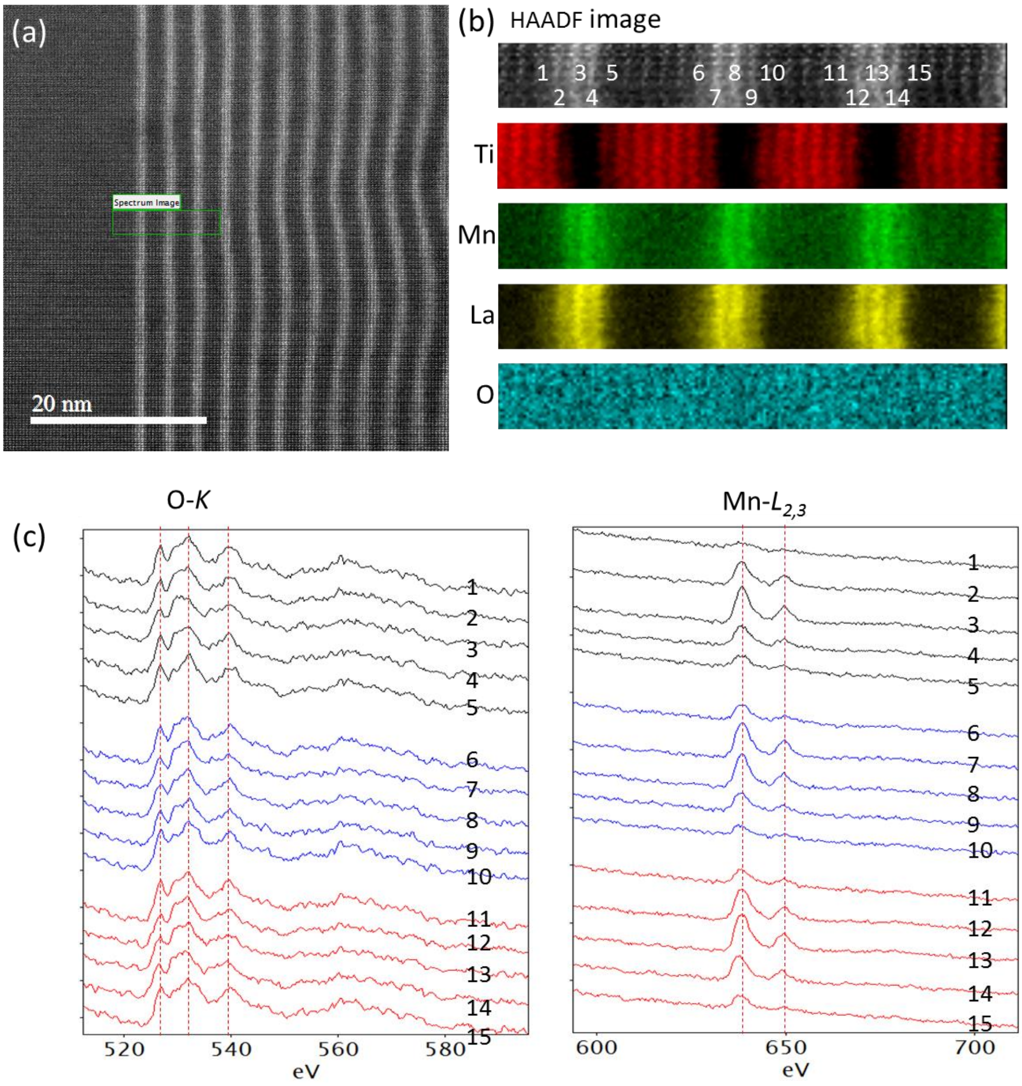


**Figure S3** EELS chemical mapping of the superlattice S15, (a) the reference STEM-HAADF image, (b) from top to bottom: corresponding HAADF image, chemical map of the Ti-$L_{2,3}$, Mn-$L_{2,3}$, La-$M_{4,5}$ and O-*K* edges. (c) EELS spectra extracted from each atomic plane (marked with a green box in the corresponding HAADF image in (a)) of the first three LSMO layers for the O-*K* and Mn-$L_{2,3}$ edges, respectively.

The chemical analyses for the first three LSMO layers are conducted in atomic detail, as shown in Fig. S2. The chemical maps extracted from the Ti-$L_{2,3}$, Mn-$L_{2,3}$, La-$M_{4,5}$, O-*K* edges respectively, are presented in Fig. S2(b), the interfaces between LSMO and STO layers are found to be sharp with a chemical intermixing limited to 1-2 atomic planes. EELS spectra showing the fine structures of the O-K edges at ~530-580 eV and the Mn-$L_{2,3}$ white lines at

~640-660 eV are extracted from each atomic plane of the three LSMO layers (Fig. S2(c)), which gives information on the Mn valence state and interfacial charge transfer. In the middle of the LSMO layers, the Mn possess a stoichiometry valence state of about ~ +3.3, however, at the interfaces crossing from LSMO to STO for 1-2 atomic planes, there is a weak shift of the Mn-$L_{2,3}$ white lines around 1eV towards lower energy indicating a valence state reduction[2] to around +2.8 with an interfacial charge accumulation. On the other hand, the chemical shift of Mn-$L_{2,3}$ is hardly been seen at the other interfaces crossing from STO to LSMO where the unexpected lattice expansion happens, indicating that the charge transfer effect is not spatially related with the interfacial structural effect. Moreover, such charge effects are too small to be observed at the corresponding O-*K* pre-edges. EELS analysis for the similar trilayer sample can be found in previous reports[3]. Such small variation of Mn valence indicating a very limited and located (1u.c.) charge transfer effect which may play a little role in the interfacial reconstruction, but certainly cannot explain the magnetic deterioration in LSMO with a thickness ranging in-between 4 u.c. and 20 u.c..

Reference

1. Koch. C. A. *Ph.D. Thesis;* Arizona State University: Arizona, U.S., **2002**.

2. Shih, S.-J., R. Sharghi-Moshtaghin, et al. *Journal of The Electrochemical Society* **2011**, 158, (10), B1276.

3. Samet, L.; Imhoff, D.; Maurice, J. L.; Contour, J. P.; Gloter, A.; Manoubi, T.; Fert, A.; Colliex, C. *The European Physical Journal B - Condensed Matter* **2003,** 34, (2), 179-192.
